# Advancing Silver Bismuth Sulfide Quantum Dots for Practical Solar Cell Applications

**DOI:** 10.3390/nano14161328

**Published:** 2024-08-08

**Authors:** Fidya Azahro Nur Mawaddah, Satria Zulkarnaen Bisri

**Affiliations:** 1 Department of Applied Physics and Chemical Engineering, Tokyo University of Agriculture and Technology, 2-24-16 Naka-cho, Koganei-shi 184-8588, Tokyo, Japan; 2 RIKEN Center for Emergent Matter Science, 2-1 Hirosawa, Wako 351-0198, Saitama, Japan

**Keywords:** colloidal quantum dots, silver bismuth sulfide, solar cells, electronic transport, nanomaterial synthesis

## Abstract

Colloidal quantum dots (CQDs) show unique properties that distinguish them from their bulk form, the so-called quantum confinement effects. This feature manifests in tunable size-dependent band gaps and discrete energy levels, resulting in distinct optical and electronic properties. The investigation direction of colloidal quantum dots (CQDs) materials has started switching from high-performing materials based on Pb and Cd, which raise concerns regarding their toxicity, to more environmentally friendly compounds, such as AgBiS_2_. After the first breakthrough in solar cell application in 2016, the development of AgBiS_2_ QDs has been relatively slow, and many of the fundamental physical and chemical properties of this material are still unknown. Investigating the growth of AgBiS_2_ QDs is essential to understanding the fundamental properties that can improve this material’s performance. This review comprehensively summarizes the synthesis strategies, ligand choice, and solar cell fabrication of AgBiS_2_ QDs. The development of PbS QDs is also highlighted as the foundation for improving the quality and performance of AgBiS_2_ QD. Furthermore, we prospectively discuss the future direction of AgBiS_2_ QD and its use for solar cell applications.

## 1. Introduction

The world faces two imminent crises threatening humanity: climate change and the energy crisis. These intertwining crises require radical solutions to develop low-energy-consuming electronic devices and to obtain energy technologies that are clean, environmentally safe, and zero-carbon in every aspect of their development. Developing highly efficient photovoltaic (PV) technologies at low production costs is one of the most promising solutions due to abundantly available primary energy (i.e., sunlight) [1]. Although the penetration of silicon solar cells has intensified in recent decades, more technological solutions are necessary to overcome the challenges related to energy conversion efficiency, the carbon footprint of current silicon solar cell fabrications, and material purity requirements, as well as the possibility of integrating them into future technologies and demands (e.g., lightweightness, compatibility with flexible electronics, among many others).

Among the emergent materials used for solar cells and other optoelectronic device applications, colloidal quantum dot QD devices are among the most prospective. Colloidal QDs are nanocrystals of such small sizes that the so-called quantum confinement effect occurs. The implications of the quantum confinement effect give rise to the size-tunable bandgap of the materials, enabling us to overcome the absorption spectral range limitation for efficient light harvesting. The 2023 Nobel Prize in Chemistry was awarded for the invention of these quantum dot concepts [2]. Furthermore, it also gave rise to the formation of discrete energy levels that essentially make them a new “giant atom” with distinct electronic and optical properties [3,4]. The existence of discrete energy levels is predicted to enable the occurrence of multiple-exciton generation (MEG), which will allow for the creation of two or more holes and electron pairs at the cost of a single photon light particle. It is essential and beneficial for developing solar cell devices with a power conversion efficiency that breaks the Shockley–Quiesser limit.

Over the past two decades, solar cells based on colloidal semiconductor quantum dots have seen significant development. Based on the 2024 NREL photovoltaic efficiency chart, perovskite QD solar cells achieved the highest efficiency, at 26.1%, and colloidal metal chalcogenide QD solar cells were not far behind [5]. In solar cell development, colloidal synthesis allows us to precisely control the dimensions and shapes of nanocrystals (NCs) and their properties [6]. The colloidal process also offers opportunities for low-cost device manufacturing through solution-based techniques, such as spin-coating, dip-coating, and inkjet printing. They can be used in roll-to-roll processing [4]. This method endows simple experimental equipment and chemicals with high-quality NCs and tunes their properties to be low cost and have low carbon footprints due to their low growth temperatures (below 200 °C) and without the demanding high vacuum process [7,8].

**Figure 1:**
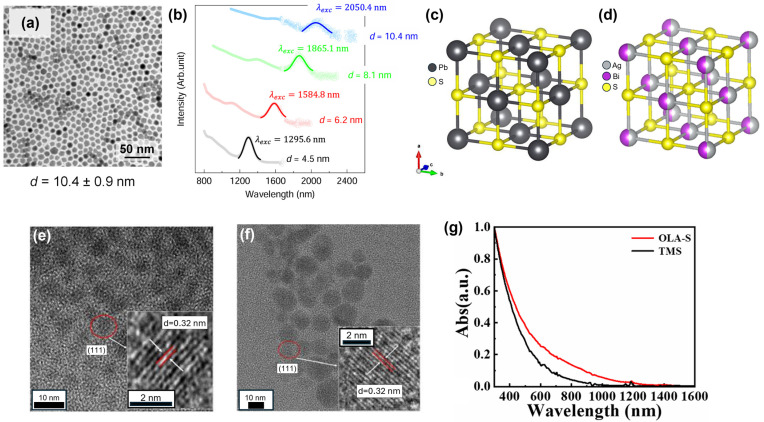
(**a**) TEM image, (**b**) absorption spectra, (**c**) crystal structure of PbS QD, (**d**) crystal structure, (**e**,**f**) TEM image of AgBiS_2_ NCs based on TMS and OLA-S, respectively, and (**g**) absorption spectra of AgBiS_2_ NC with different sulfur precursors. Reprinted and adapted from Reference [25] with permission from Nature Publishing Group and Reference [26] with permission from MDPI.

Looking at the research related to NC solar cells, Pb-, Hg-, and Cd-based semiconductor NCs (including their chalcogenides and Pb perovskite) are well-developed due to their facile synthetic accessibility and long development period [9]. Today, they show well-defined size-dependent quantum confinement effects (an example of PbS QDs is given in Figure 1a,b), and their high-performance applications in photodetectors and solar cells have been well investigated. These classes of QD compounds have become model materials in this field to understand the physical process. Although their device applications are nearly practical, the toxicity of Cd, Hg, Pb, and their compounds has become a health and environmental concern regarding their applications, particularly in several economies that have placed restrictions on their use [9,10]. This reality has motivated some researchers to start exploring the possibility of developing other NC compounds that are more environmentally friendly. Non-toxic metal chalcogenide/halides, such as ZnS [11], SnS [12], Bi_2_S_3_ [10], BiOI [13], and CuS [14], are drawing the interest of many researchers as NC materials.

Silver bismuth sulfide, AgBiS_2_ [15,16,17,18], a ternary bimetallic sulfide belonging to the I-V-VI group, has recently been introduced and reported as a promising eco-friendly material for optoelectronic applications [15,16,19,20,21]. This material has a broad absorption spectrum in the visible to near-infrared region and shows a high absorption coefficient greater than 10^5^ and 10^4^ cm^−1^ at 680 nm and 930 nm, respectively. It has a high dielectric constant and thermal conductivity, showing favorable photovoltaic characteristics. AgBiS_2_ in bulk form has a band gap of approximately 0.8 eV. By decreasing the nanocrystalline size closer to the quantum confinement regime, the band gap values can increase from 1 eV to 1.5 eV, excellently matching the Shockley–Quiesser limit requirements for solar cells [15,17,22,23]. AgBiS_2_ crystallizes in highly symmetrical crystalline structures, unlike other bismuth-chalcogenide compounds like Bi_2_S_3_. AgBiS_2_ has a pseudo-cubic rock salt structure, as shown in Figure 1d, like known nanocrystal compounds such as PbS (Figure 1c) and PbSe. This structure, which seems like a rock salt cubic structure, may enable the possible isotropic omnidirectional transport of carriers [24].

Nowadays, well-developed materials, such as Pb- and Cd-chalcogenide NC compounds, have demonstrated solar cells with a power conversion efficiency (PCE) of more than 19%. AgBiS_2_ can become the non-toxic material alternative if massive improvements and advancements in AgBiS_2_ NC synthesis can be achieved. At the start of AgBiS_2_ synthesis, SILAR and spray deposition methods produce a maximum PCE of 1.7% [20]. Utilizing the hot-injection method boosts the PCE of AgBiS_2_ NCs solar cells to 6.3% [15]. Several studies have successfully improved the efficiency of AgBiS_2_ solar cells by up to 9% [16]. There are still many unknown fundamental physical and chemical properties of this material. Several attempts have been made to increase the quality of the crystal and improve the performance of AgBiS_2_ QDs. However, to date, the morphology of this material is still far from good, as depicted in Figure 1e,f, compared to PbS QDs. The lack of proof of the size-dependent band gap feature and the absence of an excitonic peak (Figure 1g) are among the main factors that have prevented this material from exceeding the performance of Pb- and Cd-based QD materials for solar cells.

In this review, we comprehensively survey the current progress of research on AgBiS_2_ as a prospective material for optoelectronic device applications. We elaborate the development of various approaches to synthesize AgBiS_2_ NCs/QDs and functionalize them with various molecular ligands. The implications of the current approaches to synthesizing the NCs and preparing the assemblies for solar cell performances will be critically evaluated. To conclude, we will compare the state of research on this NC with the past and current research states of the more established colloidal PbS QD and other lead chalcogenides. This comparison will outline the required future research strategy for AgBiS_2_ NCs/QDs, focusing on the necessary fundamental investigation of charge carrier transport in this new emerging material system. There is a high expectation that the success story of PbS QDs can also be translated into an accelerated development of AgBiS_2_ NCs/QDs science.

## 2. Synthesis Method

The synthesis method of AgBiS_2_ QDs has evolved with the development of Pb- and Cd-based QDs. QDs are obtained by reactions between molecular precursors in organic solvent at mild temperatures (100 °C to 350 °C). The nucleation and growth of CQDs are controlled by surfactant molecules (ligands) that bind dynamically to CQD surfaces. Precise control over the stoichiometry, size, and shape of the CQDs depends on the precursors and surfactants and tuning the reaction temperature and time. Here, we recap all the methods used to produce AgBiS_2_ QDs, as shown in Table 1.

**Table 1:** Methods to obtain AgBiS_2_ QDs.

Material	Method	Precursor	Size (nm)	Application	Ref
AgBiS_2_ QDs	Chemical and sonochemical deposition	AgNO_3_, Bi(NO_3_)_3_, and Na_2_S_2_O_3_ in HNO_3_	4.2	Photoconductor	[27]
AgBiS_2_ NCs	Chemical synthesis and solvothermal	Ag(OAc), Bi(OAc), and sulfur in OA	~11	Thermoelectric	[24]
AgBiS_2_ QDs	Hot injection	AgNO_3_, Bi(NO_3_)_3_, and sulfur in OLA	8.5 ± 1.2	-	[28]
AgBiS_2_ NCs	Solvothermal	Ag(OAc), BiCl_3_, and CS_2_ in OA, CH, and 1-decanol	7.6	Quantum dot-sensitized solar cells	[29]
AgBiS_2_ NCs	Hot injection	Ag(OAc), Bi(OAc) in OA, and (TMS)_2_S in ODE	4.62	Solar cells	[15]
AgBiS_2_ thin film	Molecular precursor	AgNO_3_, Bi(NO_3_)_3_·5(H_2_O), and SC(NH_2_)_2_ in DMSO	11–67	Photoconductor	[30]
AgBiS_2_ NCs	Chemical synthesis (room temperature)	AgNO_3_ and Bi(NO_3_)_3_·5(H_2_O) in OA; sulfur powder in OCT/DDA	7–15	Photodetector	[31]
AgBiS_2_ thin films	Molecular precursor	Ag(OAc) and Bi_2_O_3_ in DTCA	~20–40	Light absorbers	[32]
AgBiS_2_ nano ink	Hot injection	Ag(OAc) and Bi(OAc) in OA; sulfur in OLA.	8.75	Photodetector	[33]
AgBiS_2_ NCs	Hot injection	Ag(iPrXa), Bi(iPrXa)_3_ in OLA and ODE	4.1–7.6	-	[34]
AgBiS_2_ QDs	Cation exchange	Ag_2_S synthesis: AgNO_3_ in toluene and OLA; St_2_S in toluene. Cation exchange: Ag_2_S QDs in toluene added by Bi(neo)_3_ in TOP	3	Solar cell	[35]

### 2.1. Sonochemical Synthesis

Sonochemistry is a method involving the formation, growth, and implosive collapse of bubbles in liquids irradiated with high-intensity ultrasound. The abrupt shrinking of these bubbles results in hot spots characterized by extreme conditions such as temperatures reaching 5000 K, pressures of 1000 atm, and rapid heating and cooling rates exceeding 1 × 10^10^ K s^−1^. Despite the reactor’s modest parameters, these localized conditions enable unique chemical reactions due to elevated temperature, pressure, and rapid cooling. Furthermore, applying ultrasonic waves to liquid–powder mixtures leads to significant alterations in the properties of the synthesized materials, known as heterogeneous sonochemical effects, which differ from those in homogeneous mediums. These effects arise from the implosive collapse of bubbles near phase boundaries, generating high-speed microjets and shockwaves. The outcome, whether particle erosion or coagulation induced by local melting, depends on the particles’ size and melting temperature. Thus, sonochemistry offers advantages such as rapid reaction rates, precise control over conditions, and the production of nanoparticles with uniform shapes, narrow size distributions, and high purity [27].

In 2008, Pejova et al. synthesized AgBiS_2_ QDs in thin film form and focused on the size-dependent structural, optical, and photoelectrical properties. Controlling the temperature of the deposition, the pH of the reaction system, and maintenance of relevant ion concentrations in the reaction system at a low level can contribute to the rate of precipitation reaction to produce thin films with decent quality and higher deposition yield. The crystal growth of AgBiS_2_ is made via a colloidal mechanism, and the reaction system is influenced by solid–liquid phase boundaries and ultrasound radiation, which affect the crystal size of the nanomaterial. The average crystal size obtained by sonochemical synthesis was 4.2 nm. Unfortunately, the shape and size distribution of the nanocrystals, or the claimed QDs, were not comprehensively evaluated using transmission electron spectroscopy (TEM).

### 2.2. Solvothermal

The solvothermal method was widely used to produce nanomaterials because tailoring the solvent, temperature reaction, reaction time, pH, and other additions can obtain a product with the desired crystalline phase, particle size, and morphology. However, this method suffers from a long reaction time that causes a low production rate [29]. Liang et al. reported homogeneous AgBiS_2_ NCs synthesized with 12 h of reaction time at 200 °C by the solvothermal method. The average size of the materials produced is about 7.6 nm, with a 2.26 nm distance between the NCs, showing the length of the oleic acid ligand. The AgBiS_2_ NCs were fabricated as counter electrodes in solar cells and compared with Pt. The obtained overall power conversion efficiency of 2.09% is comparable with Pt devices with a PCE of 1.73% under a light intensity of 100 mW cm^−2^.

In another work [24], black-colored nanocrystals of AgBiS_2_ were produced by mixing silver acetate and bismuth acetate in oleic acid for 2 h at 100 °C and placing them in a Teflon-lined stainless-steel autoclave with sulfur powder in a hot-air oven for 2 h at 180 °C.

### 2.3. Hot Injection

The hot-injection method is the most common technique to synthesize colloidal quantum dots. This method typically uses long-chain organic ligands containing amines, carboxylates, thiolates, or phosphonates to dissolve and mix the metal precursors. These ligands later function as stabilizing agents on the QD surface. The role of the ligands is not merely to be the stabilizing and solubilizing agents that prevent the aggregation of QDs. The use of ligands may also modulate some of the physical properties of the formed QDs, such as the passivation of surface defects and the reduction in nonradiative recombination [36,37]. Reflecting on the initial development of PbS and CdS QD syntheses, two protocols were most commonly applied. The first protocol typically uses metal-oxides (e.g., PbO, CdO) and (TMS)_2_S or hexamethyldisilathiane (HMS) as the metal and sulfur sources, respectively [38]. The second protocol uses chloride salts (e.g., PbCl_2_ and CdCl_2_) and elemental sulfur in oleylamine as the metal and sulfur sources, respectively [39]. These two methods have also been implemented in the AgBiS_2_ synthesis route.

AgBiS_2_ QDs were synthesized with a hot-injection method for the first time, as reported by Chen et al. [28]. Based on the purpose of obtaining monodispersed AgBiS_2_ QDs, this work demonstrates the synthesis of AgBiS_2_ QDs with a narrow size dispersion with an average diameter of 8.5 ± 1.2 nm. The shape of the QDs is faceted with a face-centered cubic (FCC) phase. Furthermore, the AgBiS_2_ QDs were made into a pellet, and the dielectric constant was measured, resulting in a high number as large as 10^5^.

Konstantatos’s group demonstrated the first AgBiS_2_ nanocrystal solar cells with a high power conversion efficiency of 6%. They developed a low-temperature hot-injection method (<100 °C) to synthesize colloidal AgBiS_2_. This work obtains the cubic rock salt structure of AgBiS_2_ NCs with diameters of 4.6 ± 1 nm. Silver acetate and bismuth acetate are used as silver and bismuth precursors to dissolve in oleic acid as a capping ligand. Hexamethyldisilathiane (HMS) is chosen as a sulfur precursor, referring to PbS and CdS sulfur sources [38,40,41]. After injecting HMS into a high-temperature mixture, the solution was allowed to cool down naturally.

**Figure 2:**
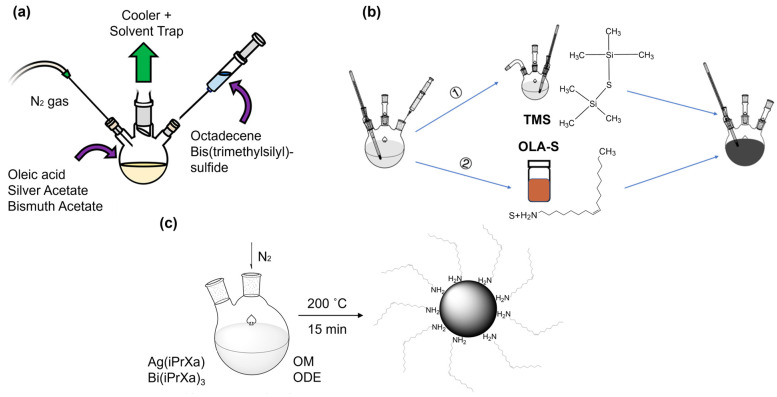
Schematic experimental setup of the synthesis of AgBiS_2_ QDs with (**a**) the hot-injection method using HMS as a sulfur precursor, (**b**) the use of different sulfur sources with TMS and elemental sulfur, and (**c**) heat-up synthesis with silver xanthate and bismuth xanthate precursors. Reprinted and adapted from Reference [17] with permission from the American Chemical Society; Reference [26] with permission from MDPI; and Reference [34] with permission from Wiley VCH.

Nakazawa et al. proposed an alternative sulfur source to replace HMS, which is expensive, toxic, stinky, and unstable in the open air in the colloidal AgBiS_2_ NCs synthesis process [33]. Oleylamine@sulfur (OLA-S) is already used in synthesizing CdS NCs [42] and PbS NCs [43]. Oleylamine, as a ligand stabilizer, can passivate surface defects and regulate NC growth [44]. Yuan et al. reported the effect of OLA-S on the growth and quality of colloidal AgBiS_2_ NCs by controlling the dosage of OLA-S, as shown in Figure 2b. OLA-S-based colloidal AgBiS_2_ NCs have a lower defect density and a broader absorption spectrum than HMS. The TEM image of AgBiS_2_ NCs based on HMS and OLA-S were depicted in Figure 1e,f, which shows no homogeneous crystal size and lattice.

Hu et al. created a modified route of the above protocol by comparing oleic acid and oleylamine as a capping ligand [45]. The Ag precursor dissolves more easily into oleylamine than oleic acid because of the amine’s complexation with the Ag cation. This modified synthesis route shows a narrower size distribution, element stoichiometry, and ligand attachment that can improve solar cell performance by being more tightly packed during film fabrication.

Another research group introduced a modified method by controlling the concentration of oleic acid in the metal precursor solution and the amount of ODE/HMS solution for use in injection [46]. Bernechea et al.’s previous synthetic route obtained irregular-sized AgBiS2 with large size distributions (5.5 ± 3.5 nm), indicating that the balance between nucleation and growth was not achieved. The modified method balances nucleation and growth by pre-adding the non-coordinating ODE solvent into the metal precursor solution and improving the PV performance up to a PCE of 5.94% [46].

The quality of AgBiS_2_ NCs/QDs resulting from the hot-injection method is still far from the state-of-the-art PbS QDs. The lattice is not well-ordered and homogeneous. However, there are still many ways to improve the lattice and uniformity of the QDs. In the latter part, we will discuss this further.

### 2.4. Room Temperature Chemical Synthesis in Ambient Atmosphere

Mak et al. pioneered a simple and fast synthesis route for AgBiS_2_ NCs [31]. Silver nitrate and bismuth nitrate pentahydrate were dissolved in oleic acid and heated at 120 °C. The sulfur precursor was prepared by dissolving sulfur powder into a long chain-length amine such as octylamine or dodecyl amine. Then, Ag/Bi and sulfur solution were mixed at a ratio of 1:1 at room temperature and atmosphere. This method resulted in cubic AgBiS_2_ without any observable by-products or oxide content. However, compared to the hot-injection method, this method could not produce nanocrystals with a homogeneous and well-ordered lattice. Nevertheless, currently it is an alternative method to obtain AgBiS_2_ at room temperature and ambient atmospheric conditions.

Konstantatos’s group also obtained AgBiS_2_ nanocrystals at room temperature and ambient atmosphere using AgI and BiI_3_ dissolved in amines [47]. A sulfur source using a sulfur–amine solution was injected into the AgI-BiI_3_ mixture, and 1-octanethiol was added. This work also produced a disordered NC lattice but showed a promising solar cell PCE of 5%. Prior to this work, the utilization of sulfur–alkylamine at room temperature was reported to be capable of obtaining large and cubic lattices of PbS NCs [48].

By following the protocols to obtain lead and cadmium sulfide that were produced via a heat-up synthesis of lead and cadmium ethyl xanthates in castor oil [49], a group reported the synthesis of AgBiS_2_ from silver and bismuth xanthates using oleylamine as a capping ligand (Figure 2c) [34,49]. Nanorod-shaped AgBiS_2_ NCs produced by tailoring the temperature can obtain different crystallite sizes.

Recently, Senina et al. proposed cation exchange as an alternative to the low-temperature synthesis of AgBiS_2_ [35]. Cation exchange is a process in which a guest cation replaces the host cation and is then incorporated into the nanocrystal structure [50]. First, Ag_2_S nanocrystals were produced by mixing silver nitrate and a bis(stearoyl) sulfide precursor in toluene and oleylamine at low reaction temperatures. Then, cation exchange was performed by adding bismuth neodecanoate to the Ag_2_S nanocrystals. The use of AgBiS_2_ nanocrystals prepared through cation exchange demonstrated a high solar cell PCE of 7.35%, showing that this synthesis approach can be an alternative to the hot-injection method, which requires high temperatures and a vacuum for prolonged periods.

### 2.5. Successive Ionic Layer Adsorption and Reaction (SILAR)

Ternary semiconductor compounds are more difficult to synthesize than binary compounds because three elements are involved, and the stoichiometry of these atoms must be precisely controlled. SILAR can be one of the options for synthesizing AgBiS_2_. Huang et al. demonstrated the growth of AgBiS_2_ sensitized on TiO_2_ photoelectrodes to produce liquid-junction solar cells [20]. In the first stage, Ag_2_S QDs were grown on the TiO_2_ photoelectrode, and in the second stage, Bi_2_S_3_ QDs were grown on top of the Ag_2_S. This resulted in a solar cell with a PCE of 0.53%.

### 2.6. Chemical Bath Deposition

Chemical bath deposition (CBD) is a low-cost method that can obtain uniformity, the feasibility of large-area preparation, and a fast growth rate. Compared to the other conventional methods, this method produces low chemical residues in the prepared films after deposition. Target ions are directly absorbed on the substrates to form thin films, and other residues stay in the solution [51]. Depositing AgBiS_2_ films with the CBD method can be performed at a relatively low temperature of 80 °C, and the film thickness can be tailored by controlling the depositing time. Nevertheless, controlling the formation of the advantageous nanostructures using this method is still challenging.

### 2.7. Synthesis by Reacting Molecular Precursor in Organic Solvent

Another versatile and flexible technique to synthesis AgBiS_2_ nanocrystals is the molecular precursor method. The conventional hot-injection method uses organic ligands as stabilizers and solvents, but they should be washed from the products many times. Meanwhile, some of the used long-chain ligands should always be exchanged with shorter ligands to enhance the performance of the materials at the application ends. These steps take time and waste organic solvents, which are substantial [52]. Therefore, the designed-molecular-precursor method can be one of the alternatives.

By mixing metal salts with certain solvents, molecular ink can be formed, resulting in films with reasonable compositional control that can be straightforwardly transferred to the printing and coating process [53]. Gu et al. mixed silver nitrate, bismuth nitrate pentahydrate, and thiourea in DMSO to produce AgBiS_2_ thin films [30]. DMSO is used because it can dissolve metal salts and produce stable molecular inks. Wu et al. fabricated AgBiS_2_ solar cells using a similar method and obtained a modest PCE of 1.4% [54].

A similar molecular precursor method developed by another research group did not use thiourea as a sulfur source. Thiourea limits the fabrication temperatures to above 250 °C and the film thickness to above 200 nm [32]. Their investigation on AgBiS_2_ solar cells revealed that they could achieve high-performance devices with low processing temperatures (100–150 °C) and a film thickness below 60 nm. This work reported the synthesis of metal–dithiocarbamate (DTC) complexes of silver and bismuth to fabricate AgBiS_2_ thin films.

Jiang et al. mentioned that the precursor method has a lower PCE than another methods, such as hot injection [52]. They emphasize the correlation between the composition of molecular precursors and device fabrication to improve the performance of AgBiS_2_ devices. The inverted p-i-n structured AgBiS_2_ photodiodes achieved a PCE of 2.04%.

## 3. Ligand Exchange

The choice of passivating ligand is one of the critical parameters for controlling QD growth by facilitating QD growth by changing the growth environment. During NC synthesis, ligand selection is crucial for tailoring the solubility and supply of active components and reducing surface energy to achieve the colloidal stability of NCs for post-synthesis processing and improving functionality [55]. Ligand exchange also overcomes the aggregation of QD due to its tiny size, resulting in controllable assembly to enhance carrier mobility. The surface ligands for QD in solid devices can affect several parameters, such as carrier density, mobility, chemical stability, charge transport, and energy band gap structure. Long-ligand passivation in QD-based devices can limit the performance by inhibiting the transfer of charge carriers. To overcome this problem, the long ligands, such as oleic acid and oleylamine, need to be exchanged with short ligands, either some organic molecules or some inorganic species such as hydroxide (OH^−^), sulfide (S^2−^), or halide (I^−^, Br^−^, and Cl^−^) ions.

In the conventional hot-injection method to produce AgBiS_2_, oleylamine and oleic acid are widely used as the solvent and capping ligand. Several studies have reported ligand exchange in the performance of AgBiS_2_ nanocrystals. The first work of AgBiS_2_ with promising results was performed by referencing many earlier works on PbS devices that showed that organic EDT molecules are suitable for replacing the oleate ligands and acting as crosslinking molecules [15]. Nevertheless, the EDT-treated AgBiS_2_ solar cells showed a poor performance, which was then attributed to the finding that thiol compounds may not adequately passivate the AgBiS_2_ NCs by looking at XPS elemental analysis and some device characterizations. Furthermore, they explored halide compounds as a more effective passivation ligand. Using iodide sourced from tetramethylammonium iodide (TMAI), the AgBiS_2_ solar cell showed a higher power conversion efficiency value of 4.8%. Together with improving the iodide-treated AgBiS_2_ solar cells, using PTB7 polymer as the hole transport layer (HTL) significantly improved their performance. The thiophene-rich polymers help to facilitate efficient charge transfer from the AgBiS_2_ and improve charge collection, resulting in efficiency values of 6.3%.

One research group compared the solar cell performance of PbS CQDs and AgBiS_2_ NCs fabricated via a solid-state ligand exchange using TMAI. After deliberately dipping the solar cell devices in deionized (DI) water for 2 min, the AgBiS_2_ NC solar cell remained highly stable without changing its chemical composition and crystallinity. On the other hand, the PbS CQD solar cells showed a decrease in PCE of 31.7% of the initial value [56]. Furthermore, they explored different surface ligands, AgI-based halometallates, as short inorganic ligands by mixing AgI and BiI_3_ with methylammonium iodide (MAI) as the ligand sources in the solid-phase ligand exchange of AgBiS_2_ NCs. This new surface ligand can replace the native oleate ligands of AgBiS_2_ NCs. Dissolving AgI in a nonpolar solvent with methylammonium iodide (MAI) will solubilize it as [AgI_2_]^−^ halometallates. The methylammonium ion acts as the counterion for [AgI_2_]^−^, helping to facilitate the colloidal stabilization of the solution-phase ligand exchange (SPLE)-prepared AgBiS_2_ NCs in the polar aprotic solvent. On the other hand, dissolving BiI_3_ and MAI in a polar aprotic solvent such as the bulky 3[MA]^+^[Bi_2_I_9_]^3−^ results in the formation of MA_3_Bi_2_I_9_ hybrid perovskite crystals and would result in incomplete ligand exchange on the AgBiS_2_ NCs.

The abovementioned method can reduce the energetic disorder and trap density of the AgBiS_2_, resulting in higher V_OC_ and PCE values of the solar cells [57]. Furthermore, to enhance the passivation on the surface of AgBiS_2_ NCs, they also introduced AgBr_2_ as an auxiliary ligand to AgI_2_ in a single-step solution phase process. The fabricated photodetector devices in the NIR region demonstrated improved performance with a high sensitivity of 1.8 × 10^12^ at 800 nm and a detectivity value of 2.6 × 10^11^ Jones at 1000 nm [18].

**Figure 3:**
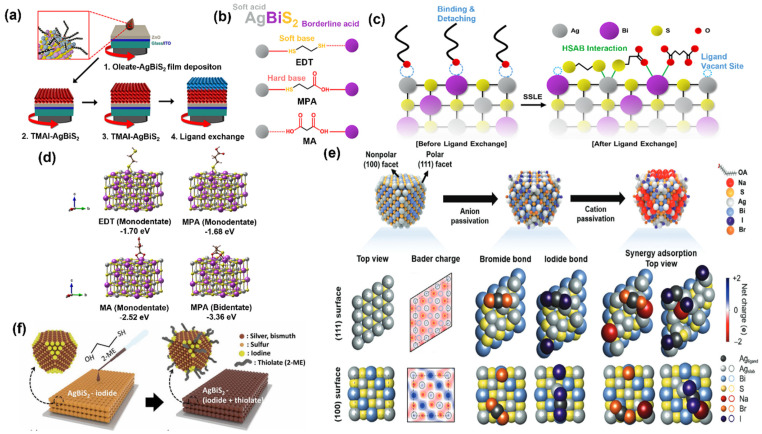
(**a**) A schematic illustration of the layer-by-layer ligand exchange process of the TMAI-treated AgBiS_2_ NC films, (**b**) a schematic explanation of the HSAB-predicted cation-selective binding preferences, (**c**) a schematic illustration of the binding action of thiol- and carboxylic acid-containing ligands onto the AgBiS_2_ NC surface, (**d**) density functional theory (DFT) of the ligand-coordinated AgBiS_2_ NC surfaces with EDT, MPA, and MA, (**e**) DFT calculations of the multifaceted passivated AgBiS_2_ CQDs from top views and visualizations of the charge density with Na, Ag, Bi, S, Br, and I ligands, and (**f**) a schematic of the iodide-capped (**left**) and 2-ME-treated (**right**) AgBiS_2_ NC films. Reprinted and adapted from Reference [59] with permission from Elsevier; and from References [16,62] with permission from Wiley-VCH.

Ming et al. also demonstrated work comparing TMAI, TBAI, BDT, ACR, thiourea, and threonine as ligands and found that TMAI provided the best performance and device stability in ambient air [58]. Therefore, TMAI passivation generally suffers from high trap-assisted carrier recombination. Other ligands such as MPA, NH_4_I, EDT, ME, MA, and iodide-based ligands were also introduced for excellent performance. The process happens between two immiscible solvent phases, one nonpolar to dissolve organic ligands and the other polar, with high dielectric constants, to effectively screen electrostatic attraction between oppositely charged ions. Bae et al. compared EDT, MPA, and MA to the ligand exchange of AgBiS_2_ [59]. Ternary NCs like AgBiS_2_ have two metal elements, which is different from binary NCs such as PbS, which have a single metal element in the NCs. It can induce unbalanced, cation-selective coordination of the molecular ligands, preventing tuning the energy levels and proper surface defect passivation. By adopting the hard–soft acid–base (HSAB) theory, MPA can function as a bidentate ligand where carboxylic acid (a hard base) binds with Bi (borderline acid) and thiol (a soft base) binds with Ag (a soft acid) on the AgBiS_2_ surface, as shown in Figure 3a–d. This work demonstrated ~12% PCE improvement over the control device. The use of MPA enables a comprehensive surface defect passivation and strong linkage along the neighboring NCs with a smaller interparticle distance, which is beneficial for charge transport properties. Another report also achieved a PCE of up to 7.3% by using MPA and methanol as ligand and solvent pairs [60]. Ammonium iodide (NH_4_I) in DMF is also used to generate the ligand exchange of AgBiS_2_ QDs [61]. The photoconductivity in thin films formed from the ligand exchange can be achieved from a single deposition step without needing multiple layer-by-layer deposition cycles. The device shows high on/off ratios and fast response times. The mild proton-donating additives during phase-transfer ligand exchange processes offer benefits in both the processing time and tuning the electronic properties of QD films. Recent work from Kim et al. utilizes a quadruple-ligand ensemble using AgI, NaI, AgBr, and NaBr, as depicted in Figure 3e [62]. These ligands homogeneously passivated the AgBiS_2_ QD surface, enhanced the PCE of the solar cell (8.11%), and shortened the response time of the photodetector. Figure 3f shows the current highest PCE of AgBiS_2_ NC-based solar cells of 9.1%, fabricated by utilizing TMAI and 2-ME, leading to the energy level optimization of NCs for efficient charge extraction.

## 4. Device Fabrication

In the early stages of the development of AgBiS_2,_ the NCs were expected to exhibit photoconductivity and favorable thermoelectric properties [27,63]. These early efforts measured the electrical conductivity and Seebeck coefficient of the synthesized materials. Some research on this material along this direction also demonstrated that the thermoelectric device can achieve a maximum ZT value of ~0.2 at 810 K [24]. Since the early demonstration of these AgBiS_2_ NCs, they were largely untouched. It was not until 2016 that some research groups started to perceive these NCs as potential materials for solar cell applications. Even so, the journey of these NCs as solar cell materials began with their use as a sensitizer or counter-electrode in dye-sensitized solar cells [15,20]. Table 2 summarizes AgBiS_2_ QD-based solar cell devices. 

The fabrication of different functional layers is essential for solar cell performance. A photon absorbed by the film produces electron–hole pairs under the built-in electronic field. The electron–hole pairs are separated and drift to the corresponding charge transport layer, such as the electron transport layer and hole transport layer [64,65]. Combining the energy levels and mobility of charge transport layers with the solid film of colloidal quantum dots (CQDs) enhances charge transport at the interface between the CQD solid film and the charge transport layer. Thus, to reduce energy losses, it is crucial to substantially decrease charge carrier recombination within the CQD solid film caused by trap-assisted recombination and interface recombination at the CQD/charge transport layer interface. This reduction aims to enhance charge carrier collection, significantly impacting CQD solar cells’ photovoltaic performance [36].

The use of an alternative hole transport layer (HTL) employing a nickel oxide with pin architecture was demonstrated by Oh et al. [66]. They fabricated p-i-n-type AgBiS_2_ NC devices following the protocols for PbS CQD-based devices. The MPA-AgBiS_2_ layer was inserted between the nickel oxide HTL and TMAI-AgBiS_2_ active layer. The results showed that adding an extra MPA-AgBiS_2_ layer improves the device efficiency by up to 5.56%. According to UPS spectra, compared to TMAI-AgBiS_2_ NC, the ligand exchange with MPA could induce a p-type character to AgBiS_2_ NC films. Combining TMAI and MPA ligands leads to efficient charge transport and extraction between ZnO and NiO layers. Furthermore, this combination induced cascade-energy level alignment that was favorable for charge carrier transport and extraction. A relatively high PCE of 9.1% was obtained by introducing 2-mercaptoethanol (2-ME) and TMAI as ligand combinations and NC/BHJ structures using BTP-4Cl [16]. Amid the use of volatile organic compounds (VOC) such as acetone and alcohol solvents can potentially cause severe environmental and health problems in the device fabrication, Ming et al. proposed the use of methyl acetate to replace VOCs and yet still resulting in decent PCE and stability of QDSCs [58].

Jiang et al. demonstrated the first work of AgBiS_2_ devices with an inverted p-i-n structure [52]. AgBiS_2_ films were stacked between the fullerene electron transport layer (ETL) and PEDOT: PSS hole transport layer (HTL). The optimization of the device was achieved by a champion device with a PCE value of 2.04%, a fill factor of 43.3%, the highest V_OC_ of 280 mV, and a J_sc_ of 16.85 mA cm^−2^. This work also showed the photodetection application by replacing the C_60_ layer with a thicker C_70_ electron transport layer to fully cover the pinholes of the thin film layer and reduce the dark leakage current and noise. The high responsivity of 0.18 A W^−1^ and a flat detectivity across the spectral range of 400 nm to 1100 nm was achieved. Furthermore, a quick response of ~700 ns was also demonstrated.

**Figure 4:**
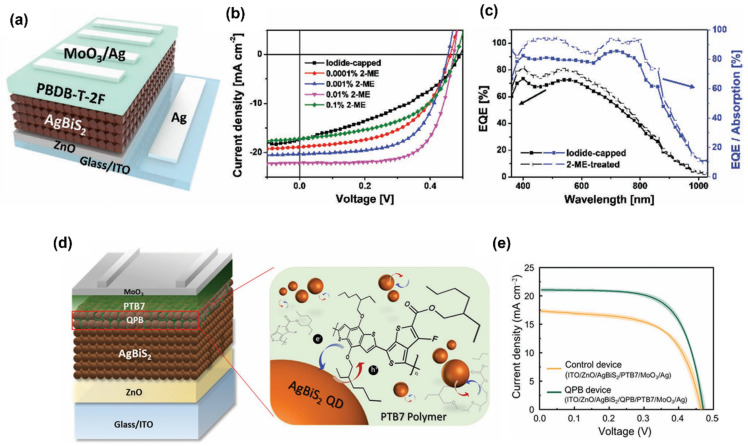
(**a**) A schematic of the AgBiS_2_ NC solar cell with a PBDB-T-2F HTL, (**b**) J-V characteristics of AgBiS_2_ NC solar cells under 1 sun illumination, (**c**) EQE and EQE/absorption spectra of iodide-capped and 2-ME-treated AgBiS_2_ NC solar cells, (**d**) a schematic illustration of the AgBiS_2_ device employing a QPB interlayer, (**e**) J-V characteristics of the control device and QPB device. Reprinted and adapted from References [16,67] with permission from Wiley-VCH.

A new design strategy was proposed to obtain an efficient energy level structure in AgBiS_2_ NC/organic hybrid solar cells [16]. Figure 4a–c shows that the researchers selected PBDP-T-2F as an HTL with a lower highest occupied molecular orbital level than PTB7, leading to increased V_OC_ of the device. Utilizing iodide and thiolate passivation also helps optimize the energy level for efficient charge extraction. Furthermore, utilizing PBDP-T-2F:BTP:4Cl achieved higher short-circuit current density through complementary absorption, obtaining the highest PCE of 9.1%.

The post-treatment of the AgBiS_2_ layer also helps to produce a high-performance solar cell device. Cation disorder homogenization under mild annealing conditions can enhance the absorption coefficient of AgBiS_2_ NCs [60]. Nevertheless, one of the problems with the current state of this AgBiS_2_ is poor charge extraction, as emphasized by Park et al. [67]. They investigated the problem of recombination at the interface by introducing a hybrid mixture of a charge acceptor and donor. A blend of AgBiS_2_ CQD and PTB7 polymers makes a quantum dot polymer bulk heterojunction (QPB) used at the HTL which increases the PCE by up to 6.78% compared to cells without QPB, as shown in Figure 4d,e. The solar cells with a well-designed glass/indium tin oxide (ITO)/SnO_2_/AgBiS_2_/PTAA/MoO_3_/Ag obtained a record efficiency of up to 9.17% (certified: 8.85%, Newport, RI, USA).

**Table 2:** AgBiS_2_ QDs-based solar cell devices.

Device Structure	Ligand	PCE (%)	V_OC_ (V)	J_SC_ (mA/cm^2^)	FF	Ref
ITO/ZnO/AgBiS_2_/PTB7/MoO_x_/Ag	TMAI	6.31	0.45	22.1	0.63	[15]
ITO/ZnO/AgBiS_2_/P3HT/Au	TMAI	4.3	0.46	16.7	0.56	[45]
ITO/ZnO/AgBiS_2_/PTB7/MoO_x_/Ag	TMAI	5.75	0.51	17.63	0.64	[56]
FTO/ZnO/AgBiS_2_/P3HT/MoO_3_/Al	No ligand exchange	1.24	0.21	15.68	0.37	[54]
ITO/ZnO/AgBiS_2_/PCE-10/MoO_3_/Ag	TMAI	4.57	0.44	18.87	0.54	[58]
ITO/PEDOT:PSS/AgBiS_2_/C_60_/BCP/Cu	No ligand exchange	2.04	0.28	16.85	0.2	[52]
ITO/NiO/AgBiS_2_/ZnO/Al	TMAI-MPA	4.74	0.46	16.46	0.58	[66]
ITO/ZnO NP/AgBiS_2_-TMAI/PTAA:C_60_F_48_/Ag	TMAI	4.4	0.44	18.5	0.56	[68]
ITO/SnO_2_/AgBiS_2_/PTAA/MoO_3_/Ag	MPA	7.3	0.45	24.9	0.61	[60]
ITO/ZnO/AgBiS_2_/PBDB-T-2F:BTP-4Cl/MoO_3_/Ag	ME	9.1	0.49	27.07	0.68	[16]
ITO/SnO_2_/AgBiS_2_/PTAA/MoO_3_/Ag	MPA	9.17	0.495	27.11	0.68	[69]
ITO/ZnO/AgBiS_2_/QPB/PTB7/MoO_3_	TMAI	6.78	0.47	21.5	0.67	[67]
ITO/ZnO/AgBiS_2_/PTB7/MoO_x_/Ag	AgX and NaX (X = Br, I)	8.11	0.54	22.33	0.67	[62]
ITO/SnO_2_/AgBiS_2_/PTAA/MoO_3_/Ag	MPA	7.35	0.486	23.81	0.64	[35]

## 5. Comparison to PbS QD Development

The PbS colloidal QD is one of the current model materials in the field, together with the other Pb-chalcogenide, Cd-chalcogenide, and Hg-chalcogenide compounds. Together with the lead-perovskite QDs, PbS QDs lead the development of emerging solar cells. By comparing the research states in the PbS colloidal QD, we will obtain strategic pathways for further developing AgBiS_2_ QDs as viable solar cells and optoelectronic materials.

**Figure 5:**
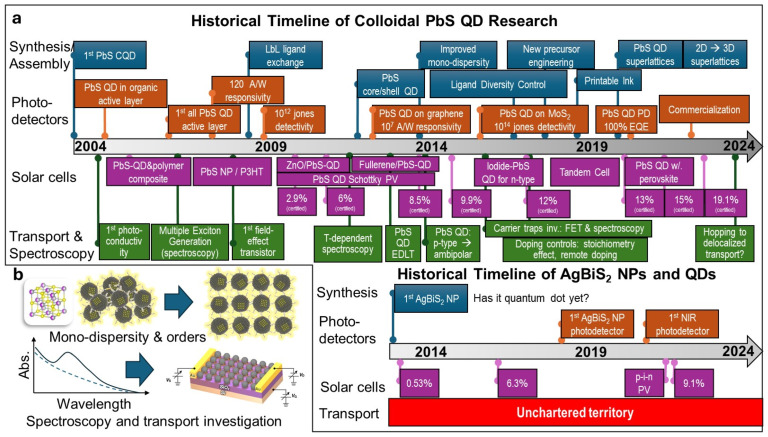
(**a**) Comparison of the historical timeline of the research and development of PbS colloidal quantum dots for optoelectronic devices (photodetectors and solar cells) and the current research stage of AgBiS_2_ nanoparticles and nanocrystals. (**b**) Among the most urgent investigations that should be undertaken are how to improve the monodispersity of the nanocrystals, to establish methods to create ordered assemblies, clarify their quantum confinement properties through spectroscopy, and investigate the charge transport process in their assemblies, including using field-effect transistors as a tool. Components are adapted from Reference [102] with permission from the American Chemical Society.

Pb-based QDs were developed more than two decades ago (Figure 5a) [38], by the optimization of stoichiometry, precursors, and ligand selection, resulting in high-quality crystal PbS QDs [39,70,71,72,73]. Numerous studies related to charge carrier transport and the improvement of device structures have successfully obtained high-performing PbS QD-based optoelectronic devices [25,74,75,76,77,78,79,80]. Until now, the progress of the PbS QD has already moved toward commercialization and the exploration of new applications [81,82,83]. The National Renewable Energy Laboratory (NREL) chart shows the development of the power conversion efficiency of QD-based solar cells, starting from the ZnO/PbS solar cells achieving 2.9% in 2010 [84], until the recent result showing an efficiency value of more than 19% in Pb-based perovskite QD solar cells [85]. This recent device still achieves a high performance with 18.1% efficiency even after 1200 h, confirming the stability of this material.

The first synthesized PbS by Hines et al. in 2003, using the hot-injection method, sparked the exploration of Pb-chalcogenide QDs [38]. This work utilized lead oxide (PbO) dissolved in oleic acid and HMS in ODE or TOP. Another precursor of PbCl_2_, OLA, and sulfur can also produce high-quality PbS QDs [86]. The variation ratio of PbCl_2_ and sulfur can control the particle size. Ma et al. proposed a one-step strategy utilizing PbI_2_ and N, N-diphenyl thiourea (DphTA) in DMF as lead and sulfur precursors [87]. These QD inks can be directly used for device fabrication without ligand exchange. This method performs more simply and cheaply than conventional methods, such as hot injection.

Long-chain ligands, such as OA, must be exchanged with short-chain ligands to obtain QD films with low trap density and high mobility. In 2006, for the first time, organic-solvent-based ligand exchange was used to replace 2.5 nm OA with n-BTA with a length of 0.6 nm in photodetector devices [88]. These devices show a significant photoconductivity gain with a responsivity greater than 10^3^ A W^−1^, surpassing the detector made of epitaxially grown InGaAs at that time. Further, continuous development has been updated to use the other organic ligands (e.g., MPA, EDT, BDT). The first observed photovoltaic effect of PbS QDs was reported in 2005 [89]. In 2008, the first PbS QDs Schottky diode and photodetector were demonstrated [90]. At the same time, Luther et al. reported that processing p-type QDs using EDT ligands significantly improved the electronic properties of PbS QDs [91]. However, the PbS-EDT still demonstrated a low carrier mobility. Therefore, to improve the responsivity of the device, Konstantatos et al. introduced PbS QDs on top of ultra-high-mobility graphene [92]. Since then, the combination of QDs with a broad range of materials has started to be investigated.

Understanding the fundamental mechanism of charge carrier transport in the assembly of QDs is crucial to improving solar cell performance. One way to investigate the charge carrier transport in the colloidal QDs is using field-effect transistors (FETs). Since 2011, some early efforts have been made to measure charge carrier mobility values employing solid gate FETs [93] and through electric double-layer FET (EDLT) that utilizes electrolytes for gating [76,94]. From FET studies, many parameters related to charge carrier transport under different values of charge densities can be elucidated. Nevertheless, the first thing that can be confirmed is the charge carrier types involved in the transport process. For a long time, PbS QD has been perceived as a p-type semiconductor with a modest value of hole mobility (<10^−3^ cm^2^ V^−1^ s^−1^) [93,95]. Meanwhile, many of the EDLT studies showed ambipolar transport. It was not until 2014 when Balasz et al. clarified that the perception of PbS QD acting as a p-type semiconductor was a result of the oxygen absorption in the QD assemblies that deeply trap the electrons, while intrinsically, PbS QD is an ambipolar material that can support both electron and hole transport [77]. This realization enables the understanding of why thin PbS QD Schottky-type solar cells can be highly efficient [96].

Since the PbS QD is known to be ambipolar, and with proper care to avoid oxygen and moisture, the electron mobility has typically been higher than the hole mobility; various strategies to tune the charge carrier types have been attempted [97]. These attempts include using various ligands and doping on the surface of the QDs. Replacing the ligands on the surface of the PbS QDs not only shifts the Fermi level but may also shift the conduction and the valence energy values [98]. One of the origins is the interplay between the doping process and trap passivation. Since trap levels also play a significant role in controlling the electronic properties of the PbS QDs, quantifying the trap density and their energetics is essential. FET studies provide a robust platform for such investigation, mainly when two different gate structures are utilized [99,100,101]. FETs and EDLTs were also used to test the changes in the electronic structure of PbS QDs when different ligands replaced the long oleic acid [76,102,103,104], or when different kinds of inorganic ligands, such as halide ligands, were also used [105,106]. Using the sulfuric-based ligand may alter the stoichiometry of the QDs, resulting in a shift of their Fermi level. Measuring FET and EDLTs can quantify this shift, where excess sulfur leads to intrinsic p-type behavior [70]. The use of some thiophene-based organic ligands can also achieve similar behavior [102].

The structure of the FET and EDLTs, where the charge carrier flows in the planar direction, allows the direct correlation between the QD assembly structures and the resulting charge carrier transport properties. It is difficult to achieve in the buried interface of many other electronic device architectures. Therefore, attempts have been made to correlate how the assembly engineering in the ligand-exchanged QD assemblies will give rise to different values of obtained holes and electron mobilities. Various kinds of assembly techniques can lead to varying levels of assembly orders [107]. Furthermore, this type of research stimulates the explorations of obtaining large-scale highly ordered QD assemblies in superlattices. Novel techniques were recently invented, including selective ligand stripping [108], and several different processes to obtain epitaxially connected or nearly-epitaxially connected superlattices [25,109,110,111,112]. Improvements in electron and hole mobility values by several orders of magnitude through the perfection of the QD superlattice formations were recently reported. Furthermore, there is much more substantial proof that delocalized charge carrier transport through miniband formation in the highly-ordered colloidal QD superlattices may be achieved [25,111,113]. The consequential significance of the recent results of charge carrier transport improvements through FET/EDLT device investigations has yet to be realized. This research pathway may significantly enhance solar cell performance and fulfill much of the unrealized promise of colloidal PbS QD materials.

Intensive spectroscopic studies to probe the charge carrier transport in PbS and Pb-chalcogenide QDs are also an essential branch of the field, which have been able to elucidate the dynamics of charge trapping behavior and the relationship between structures and the charge carrier diffusion time and length. Various spectroscopic efforts have been performed, including ultrafast time-resolved photoluminescence [114], pump–probe spectroscopy [115,116,117], and spectroelectrochemistry [118,119,120], as well as microwave conductivity [121,122]. The intensive efforts of spectroscopy studies and device studies have enabled the relatively comprehensive knowledge of the PbS and Pb-chalcogenide QDs as present and future solar cell materials, which can also become the template for the investigations of many other colloidal QD compounds that are still in their early stages, like AgBiS_2_, or have still not been invented yet.

## 6. Conclusions and Future Outlook

In this review, we have introduced AgBiS_2_ QDs as an alternative environmentally friendly CQD for solar cell application. Further, we summarized all the existing synthesis methods, ligand choice, and device fabrications of AgBiS_2_ QDs. The development of this material successfully achieved a high efficiency of 9%. However, the result for Pb- and Cd-based QD materials is still far from that of Pb- and Cd-based materials. The development of AgBiS_2_ CQD solar cells remains challenging because of the limitation of surface defect passivation and band alignment [36]. In addition, we discussed the development of PbS QD materials as the foundation for the future outlook of AgBiS_2_ QDs. By looking at the development of PbS, several steps can be used to improve the performance of AgBiS_2_ QDs (Figure 5b). Improvements in crystal quality, electronic properties, transport studies, stability, and device architectures are needed to obtain high-performance AgBiS_2_ QD-based solar cells.

- Improving the orders of the nanocrystal assemblies

In order to achieve high PV efficiencies, monodisperse CQDs with a narrow size distribution are required so that the device V_OC_ mismatch to their bandgap can be reduced [123]. The quality of CQDs is determined by the perfection of the cores’ crystals, the thorough passivation of their surfaces, and the homogeneity of the crystal size and shape. High uniformity is crucial to reserve the near-discrete character of the density of states for an ensemble of CQDs [124]. One feasible method to control AgBiS_2_ solar cells’ band gap and light absorption spectrum is tailoring the QD synthesis protocols at a high temperature to obtain slightly larger diameter with a smaller band gap value [36,125]. Compared to well-developed QDs such as Pb- and Cd-chalcogenide, AgBiS_2_ still lacks uniformity which is critical for forming nanocrystal superlattices with enhanced charge carrier conductivity.

Several enhancements through synthesis protocols can be used to obtain uniform and well-ordered arrangements. Various colloidal synthesis protocols can be explored, such as the conventional hot-injection method, one-pot synthesis, and room-temperature chemical synthesis [12,17,46]. The solution-based colloidal method is more promising since it allows controlled sizes and shapes with a broad spectrum of colloidal semiconductor NCs [55]. First, precursor selection is made, and precursors with high purity and controlled reactivity are chosen to obtain uniform and high-quality nanocrystal formation. For example, the sulfur precursors for metal chalcogenide synthesis should always be organo-sulfur. A viable alternative should also be found. Second, temperature and solvent optimization must be conducted. Tuning the temperature and choice of solvent can control the size, shape, and crystallinity of AgBiS_2_ NCs. In the PbS synthetic process, PbO-TMS-OA was modified by diluting OA in ODE and using n-trioctylphosphine (TOP) as the co-ligand to improve the size distribution, stability, and passivation of the ligands [126,127]. Third, capping ligands can prevent agglomeration and control the surface chemistry that affects electronic and optical properties. One critical factor deciding power efficiency in solar cells is the quality of charge carrier transport. Stronger electronic coupling can improve charge carrier mobility [128].

- Improving Electronic Properties

One feature that is interesting about QDs is the size-dependent band gap that is related to the quantum confinement effect. Tailoring the bandgap of AgBiS_2_ NCs by size control or doping can improve light absorption and photovoltaic performance. A type-I core-shell AgBiSe_2_/AgBiS_2_ increases the PCE from 1.4% (AgBiS_2_) to 2.1% (AgBiSe_2_/AgBiS_2_) [129].

Transistor studies are necessary to understand the fundamental mechanism of the electron transport role where charge carrier densities can be high and influence the optoelectronic properties. Improving the charge carrier mobility in FETs is vital to increase the potential for more broad applications of AgBiS_2_ NCs. One of the ways to improve the charge carrier mobility is by doping material chemically [130]. Doping the QDs can shift the Fermi level, increase solar cell device efficiency, and improve the mobility of transistor devices. As far as we know, no transistor study has been performed for AgBiS_2_ NCs.

The study of photoconductivity also plays a crucial role in developing advanced optoelectronic devices, such as photodetectors, solar cells, LEDs, and sensors. It can help us to understand carrier dynamics, including the generation, separation, transport, and recombination of electron–hole pairs. Among many other spectroscopy techniques, time-resolved microwave conductivity (TRMC) can be used to understand the charge carrier dynamics [131].

- Enhancing Light Absorption and Photocurrent

One of the essential properties to be sought in CQDs is the existence of an excitonic absorption peak. The existence of excitonic peaks would play a crucial role in understanding the optoelectronic properties of QDs since it is one of the most apparent direct definitions of the quantum confinement effect. However, to date, a clear excitonic peak has never been found in all AgBiS_2_ NCs/QDs reports. There are many possible reasons behind that, such as the non-uniform size of the QDs, the existence of surface defects, aggregation, and the band gap mismatch due to size variations. If a clear excitonic peak can appear, it will enable us to enhance the performance of diverse kinds of device applications. Investigating the excitonic peak will lead to a clue towards obtaining a high-quality crystal size and shape uniformity. In the early days of the small-sized CdSe QDs case, researchers first obtained the “hidden” excitonic properties of the second and third electronic transition states of broadened absorption peaks [132].

QD-based solar cells have the potential to show higher power conversion efficiencies through enhanced multiple exciton generation (MEG), which can be the manifestation of shape–size control, internal QD heterojunction interface formation, and many other parameters related to material compound exploration. MEG can boost the photocurrent where single high-energy photons generate multiple electron–hole pairs. MEG appears when excitons with energy exceeding twice the band gap energy relax to the band edge by creating an additional electron–hole pair via impact ionization. MEG is anticipated to be more efficient in quantum-confined nanostructures than in bulk semiconductors because the requirement for momentum conservation is relaxed, and carrier–carrier interactions are enhanced due to strong confinement within the nanostructure [133].

Time-resolved photoluminescence measurement can be used to study the ultrafast dynamics of excitons in QDs, including carrier relaxation, recombination, and energy transfer processes occurring on femtosecond to nanosecond timescales. In order to improve the PCE of AgBiS_2_-based solar cells, a photoluminescence study can help us to identify and characterize them [134]. Furthermore, introducing many other nanostructures into the solar cells may enhance the photocurrent, such as the enhancement of light absorption via localized surface plasmon resonance.

- Device Architecture Sophistication, Stability, and Environmental Compatibility

Improving material components and device architectures would enable rapid advancements in CQD solar cell development. The exploration of novel device structures (e.g., tandem or multijunction devices) to maximize light harvesting and improve efficiency must be accelerated for these AgBiS_2_ NCs. Nevertheless, the oldest solar cell fabrication, Schottky solar cells, can also be revisited to evaluate the size-tuned band positions of different CQD films relative to the known work functions of metals and elucidate the roles of drift, diffusion, and depletion in AgBiS_2_ CQD-based devices. This architecture can investigate the actual performance of AgBiS_2_ QDs without any HTL or ETL. More variations in heterojunction architecture can also be developed to improve charge extraction. Fundamental research efforts to optimize the interfaces between AgBiS_2_ NCs and other materials can reduce losses and improve charge extraction.

AgBiS_2_ QDs are still in the early stage of exploration. Improving the crystal quality, electronic properties, and light absorption, as well as achieving a quantum confinement effect, can be the next step in obtaining a high performance for solar cell devices. Investigation of the stability also enhances the potential application of this material. Fabricating device-efficient architecture is also worth trying for sufficient solar cell devices. Developing new materials is indeed a challenging issue. Many properties require in-depth investigation, and it is worth noting that having a “green” new technology for many applications with a high performance can be achieved. Further investigation does not merely assess the stability of AgBiS_2_ QDs but also explores more potential applications of this material.
